# Metabolism during anaesthesia and recovery in colic and healthy horses: a microdialysis study

**DOI:** 10.1186/1751-0147-51-10

**Published:** 2009-03-10

**Authors:** Anna H Edner, Birgitta Essén-Gustavsson, Görel C Nyman

**Affiliations:** 1 Department of Clinical Sciences, Faculty of Veterinary Medicine and Animal Science, Swedish University of Agricultural Sciences, Uppsala, Sweden; 2 Department of Medical Sciences, Clinical Physiology, University hospital, Uppsala, Sweden

## Abstract

**Background:**

Muscle metabolism in horses has been studied mainly by analysis of substances in blood or plasma and muscle biopsy specimens. By using microdialysis, real-time monitoring of the metabolic events in local tissue with a minimum of trauma is possible. There is limited information about muscle metabolism in the early recovery period after anaesthesia in horses and especially in the colic horse. The aims were to evaluate the microdialysis technique as a complement to plasma analysis and to study the concentration changes in lactate, pyruvate, glucose, glycerol, and urea during anaesthesia and in the recovery period in colic horses undergoing abdominal surgery and in healthy horses not subjected to surgery.

**Methods:**

Ten healthy university-owned horses given anaesthesia alone and ten client-owned colic horses subjected to emergency abdominal surgery were anaesthetised for a mean (range) of 230 min (193–273) and 208 min (145–300) respectively. Venous blood samples were taken before anaesthesia. Venous blood sampling and microdialysis in the gluteal muscle were performed during anaesthesia and until 24 h after anaesthesia. Temporal changes and differences between groups were analysed with an ANOVA for repeated measures followed by Tukey Post Hoc test or Planned Comparisons.

**Results:**

Lactate, glucose and urea, in both dialysate and plasma, were higher in the colic horses than in the healthy horses for several hours after recovery to standing. In the colic horses, lactate, glucose, and urea in dialysate, and lactate in plasma increased during the attempts to stand. The lactate-to-pyruvate ratio was initially high in sampled colic horses but decreased over time. In the colic horses, dialysate glycerol concentrations varied considerably whereas in the healthy horses, dialysate glycerol was elevated during anaesthesia but decreased after standing. In both groups, lactate concentration was higher in dialysate than in plasma. The correspondence between dialysate and plasma concentrations of glucose, urea and glycerol varied.

**Conclusion:**

Microdialysis proved to be suitable in the clinical setting for monitoring of the metabolic events during anaesthesia and recovery. It was possible with this technique to show greater muscle metabolic alterations in the colic horses compared to the healthy horses in response to regaining the standing position.

## Background

Microdialysis as a means to repeatedly sample and analyze various substances in the interstitial fluid and in body cavities has enabled the study of local tissue metabolic events [1-7]. The great advantage with this technique is that it allows real-time monitoring of the metabolic events in local tissue with a minimum of trauma. When introduced into the tissue, the microdialysis catheter acts as an artificial blood capillary where the perfusion fluid in the catheter equilibrates with the concentrations of water-soluble substances in the extra cellular fluid [8,9]. Commonly assessed substances for studying metabolic alterations in tissues are lactate, pyruvate, glycerol, glucose, and urea.

Lactate and pyruvate play a central role as metabolic markers in ischaemia research and with increasing frequency these are studied using microdialysis [6,10,11]. Our group has used the microdialysis technique and sampling of muscle biopsies and found that anaesthesia in healthy horses was associated with an increased production of muscle lactate and decreased content of ATP indicating anaerobic metabolism [12,13]. This may be related to general or local hypoperfusion [14-16].

Increased plasma lactate concentrations are frequently measured in colic horses subjected to emergency abdominal surgery [17-19]. Muscle biopsy data have shown increased muscle lactate levels during anaesthesia in colic horses [20]. However, there is limited information about muscle metabolism during the early recovery period and thus the hypothesis was that microdialysis could be a suitable technique for studying muscle metabolic events during anaesthesia and recovery in healthy and colic horses.

The aims were to evaluate the microdialysis technique as a complement to plasma analysis and to study the concentration changes in lactate, glucose, glycerol, and urea in both colic and healthy horses, during anaesthesia and up to 24 h after standing.

## Materials and methods

### Study design

The Ethical Committee on Animal Experiments in Uppsala, Sweden approved the research protocol. The study period comprise the time from before anaesthesia until 24 h after recovery to standing.

The material presented below is part of a larger study investigating metabolic changes in plasma and muscle biopsy specimens up to seven days after recovery from anaesthesia, in 20 colic horses subjected to emergency abdominal surgery as opposed to in 20 healthy horses subjected to prolonged anaesthesia in dorsal recumbency [20]. The present study comprise 10 of the colic and 10 of the healthy horses that, in addition to plasma and muscle biopsy sampling, were subjected to muscle microdialysis. Colic horses entered the present study when microdialysis was performed and where samples were obtained at least during anaesthesia and in to recovery. The 10 included healthy horses were those anaesthetised during 2000.

### Horses

#### Colic horses

Ten client-owned colic horses (C) subjected to acute abdominal surgery at the horse clinic at the Swedish University of Agricultural Sciences, from January to April 2001 and from January to June 2002 were studied. The horses were referred by field practitioners or smaller equine clinics because of unresolved acute colic of different genesis. On arrival at the university all horses were examined clinically and treated medically and later surgically by the veterinarian on duty. The approximate duration of colic (and withdrawal of food) from observation of signs until time of surgery in the sampled horses varied from 6 h up to 2.5 days with a median of 24 h.

#### Healthy horses

Microdialysate and plasma samples from 10 healthy, Standardbred, research horses (H), anaesthetised in dorsal recumbency for participation in two other anaesthesia research projects were used for comparison of results. These horses were owned by the former Department of Large Animal Clinical Sciences, SLU, Uppsala, Sweden and were housed at the department where they were outdoors during the day and stabled at night. They were fasted for 12 h before anaesthesia.

A summary of details regarding age, sex, breed and weight of all horses are shown in Table 1.

**Table 1:** Summarised data on the 10 colic and 10 healthy horses included in the present study

	**Colic horses**	**Healthy horses**
Number of horses:	10	10

Age: mean (range)	10 (3–15) years	7 (4–17) years

Sex:	4 mares, 5 geldings,1 stallion	5 mares, 5 geldings

Breed:	1 Shetland pony, 2 Standardbred trotters, 1 Arabian, 6 Warmblooded riding horses	10 Standardbred trotters

Weight: mean (range)	520 (230–695) kg	503 (428–584) kg

The mean values for age, breed and weight (kg) of the horses are given with the range within the parenthesis.

### Anaesthesia

#### Colic horses

The procedure has been described previously [20] and is only described briefly below.

In horses in which additional sedation or analgesia before induction was necessary, this usually consisted of an alpha-2 agonist and butorphanol. In eight horses, anaesthesia was induced with an intravenous (IV) infusion of 7.5% guaifenesin to effect and a bolus dose of 3.1–4.4 mg/kg thiopentone sodium. Diazepam (0.02 mg/kg IV) and ketamine (2.2 mg/kg IV) or guaifenesin and ketamine (2.1 mg/kg IV) were used for induction in two horses. The horses were intubated and anaesthesia was maintained with isoflurane in oxygen delivered by a semi-closed large animal anaesthetic circuit with horses in dorsal recumbency. In five horses breathing was spontaneous while in five horses intermittent positive pressure ventilation (IPPV) was instituted for most or part of the procedure. Cardiovascular and respiratory function was monitored with standard techniques.

Intravenous, isotonic electrolytes were given to all horses. Hypotension (mean arterial pressure <70 mmHg) was treated with an IV infusion of a dextran colloid or dobutamine (0.5–2 μg/kg/min) or both. After anaesthesia and abdominal surgery the horses were allowed to recover in a padded box and supplemented with oxygen insufflated at 15 L/min through the tracheal tube or the nostril. Treatment in the recovery box was provided as judged from case to case by the treating veterinarian but xylazine and flunixin were given to most horses.

#### Healthy horses

The healthy horses were premedicated with detomidine (10 μg/kg IV) 10 min before intravenous induction with 7.5% guaifenesin to effect and a bolus dose of thiopentone sodium (4.5 mg/kg IV). Intubation and maintenance of anaesthesia was as described above. Fluid therapy consisted of isotonic electrolytes at 4 mL/kg/h. In one horse breathing was spontaneous, four horses were ventilated with IPPV for the whole procedure, and five horses experienced both modes of ventilation. After anaesthesia the horses were allowed to recover in a padded stall as described above. Six horses were given xylazine (0.15 mg/kg) and flunixin (1.1 mg/kg) IV after discontinuation of inhalation anaesthesia. No recovery assistance was given.

### Post anaesthesia

Medical treatment during the 24 h-study period after recovery to standing was provided at the distinction of the treating veterinarian as judged necessary by the horse's condition. All surviving colic horses were given IV fluids, antibiotics (penicillin or gentamicin or both) and flunixin. Other analgesic drugs provided were alpha-2 receptor agonists, dipyrone, pethidine, and butorphanol. An IV infusion of glucose (2.5%) was given to one horse (C1). The healthy horses received medical treatment only if complications developed.

No feed was provided to the colic horses during the study period. The healthy horses were provided water and hay (approximately 8 kg/day) and a wet mixture consisting of beet pulp, wheat and barley bran (0.5–1 kg/day) when they were alert after recovery from anaesthesia, approximately after 4 hours.

### Samples

#### Sampling and analyses of dialysate

**Figure 1:**
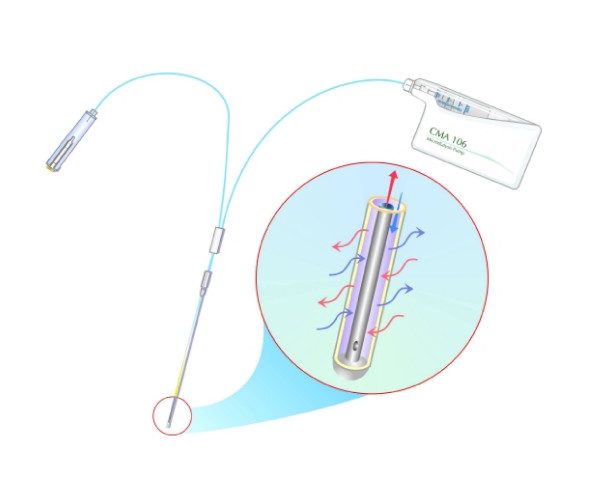
**An illustration of the microdialysis catheter and infusion pump**. The microdialysis catheter consists of a 600-mm-long inlet tube, a 90-mm-long double-lumen tube, and a 220-mm-long outlet tube to which the microvial is fastened. The double-lumen tube has a 60-mm-long shaft (0.9 mm in diameter) and a 30-mm tip (0.6 mm in diameter) where the outer layer consists of a polyamide dialysis membrane. The perfusate enters the catheter between the inner tubing and the outer dialysis membrane, allowing for the process of dialysis, the dialysate is subsequently transported away inside the inner tube to be collected in the microvial. The illustration was published with kind permission of CMA/Microdialysis AB, Solna, Sweden.

After placing the horse in dorsal recumbency on the surgery table, the horse was slightly tilted to the right and a commercially available microdialysis catheter (CMA 70 Brain Microdialysis Catheter, CMA/Microdialysis AB, Solna, Sweden) (Figure 1) was introduced into the left gluteal muscle through a custom-designed split catheter. A small, battery-powered infusion pump (CMA 106 Microdialysis pump, CMA/Microdialysis AB, Solna, Sweden) was secured to the horse's tail with self-adhesive wrap and protected with plastic. Using this pump a modified Krebs-Henseleit buffer, with the addition of a colloid (40 g/L dextran-70), was perfused through the microdialysis catheter at a perfusion rate of 0.3 μL/min. This means that the concentration of the recovered substance in the dialysate is very close to the true interstitial concentration of that substance (a relative recovery of glucose of 90% and that of lactate approximates 100% in humans) [8,21]. A stabilisation period of 90 min was allowed after insertion of the catheter before beginning to collect the first sample, subsequently referred to as dialysate. Samples were collected continuously in 20- to 40-min sequences during anaesthesia and when possible during recovery. After recovery to standing, sampling continued in 30- to 60-min sequences for 2–3 h and thereafter in 1–3-hour sequences for as long as the catheter was functioning up to 24 h. Every vial was weighed before and after sampling to allow estimation of fluid loss or gain. The vials were kept in protective vials on ice for 10–20 minutes before being weighed, put into tight plastic bags and frozen at -20°C until analysis. The dialysate was analysed for its concentrations of lactate, glucose, urea, and glycerol with enzymatic colorimetric methods using a commercially available sample analyzer (CMA/600, CMA/Microdialysis AB, Solna, Sweden). In five colic horses pyruvate was analysed instead of glycerol. Each horse's sequence of samples was analysed at the same time to decrease the within-horse variation.

#### Sampling and analyses of blood samples

Venous blood was sampled in the awake state before induction; at every hour of anaesthesia; at 15 minutes and at every hour after discontinuation of inhalation anaesthesia whilst still recumbent; at 15 and 30 min, 1, 2, 4, 8, 12, and 24 h after standing. The blood samples were collected from a catheter in the jugular vein. Samples for assays of plasma lactate, glycerol, glucose, and urea were taken in heparinised vials. Samples were kept on ice until they were centrifuged (within 30 minutes) and stored at -80°C until analysed. Plasma lactate was assayed with a lactate analyser (Analox GM7, Analox Ltd, London, Great Britain). Glycerol was determined using a commercial kit (EnzyPlus, Diffchamb AB, Västra Frölunda, Sweden). Glucose was assayed using modified fluorometric methods [22]. Urea was determined by a spectrophotometric method using standardised reagent kits (Konelab 30, Kone Instruments, Espoo, Finland).

#### Statistical analysis

Statistical analyses (Statistica 6.0 and 7.0, StatSoft^®^, Inc. Tulsa OK, USA) of the microdialysate results were performed on the following samples: the last sample obtained during anaesthesia, the sample obtained during the horse's successful attempt to reach the standing position (sample 0), the samples obtained 1 h and 2 h after standing, and also the sample representing the mean maximum change (increase or decrease) from the end of anaesthesia was seen. The timepoint for this sample could be different in individual horses. No statistical analysis was performed on the temporal changes in dialysate during anaesthesia due to the different duration of anaesthesia between horses. Statistical analysis beyond 2 h after standing was not performed.

Statistical analyses of blood sample results were performed on the sample obtained before anaesthesia, on the first and last samples taken during anaesthesia, a mean of the samples taken during recovery from anaesthesia when still recumbent, 15 minutes and 1 h and 2 h after regaining the standing position.

Temporal changes and differences between groups were analysed with an ANOVA for repeated measures followed by Tukey Post Hoc test or Planned Comparisons when the sphericity assumptions were violated. If the interaction Group*Time was significant, simple effects were examined, i.e. effects of one factor holding the other factor fixed. The p-values were then corrected according to the Bonferroni procedure. The distribution of dialysate glucose was skewed and was log transformed before formal analyses. In all analyses, a p-value of <0.05 was considered significant. Dialysate and plasma results are reported and shown in the figures as means ± standard error of means (SEM).

For the statistical analyses, the plasma sample taken at 15 minutes after standing was compared to the dialysate sample collected when the horse regained the standing position (0). In the graphs, these two samples are the point of synchronisation. Since the horses spent different lengths of time lying down in recovery, the samples before time 0 may for different horses represent samples obtained either during anaesthesia or samples obtained after termination of inhalation anaesthesia when still recumbent.

Samples from two colic horses (C8 and C14) were not included in the statistical analyses and are also discussed separately since these horses were judged to be in a worse condition as interpreted from their pre-operative status. The glucose values from the horse (C1) receiving glucose were excluded from statistical analysis.

## Results

### Anaesthesia and outcome

The mean (range) duration of anaesthesia was 208 (145–300) minutes for the colic horses and 230 (193–273) minutes for the healthy horses. The mean (range) time from discontinuation of anaesthesia until the standing position was regained was 52 (15–105) minutes in the colic horses and 53 (18–75) minutes in the healthy horses. Eight colic horses needed one or two attempts to stand. Two colic horses (C8, C14) never regained the standing position. The quality of recovery for those horses that regained the standing position was mostly good, it was violent in one horse (C13) and another horse (C15) did some paddling before regaining the standing position. Both of these horses had signs of slight hind limb dysfunction for one day. Seven of the ten colic horses survived at least 24 h after recovery to standing. One horse (C8) died from cardiovascular collapse and pulmonary oedema 65 min after termination of inhalation anaesthesia without ever making any attempts to stand or lie in the sternal position. One mare (C14) was in severe pain and had spontaneous reflux of gastric contents and metabolic acidosis (BE: -17) in the recovery box. She made one assisted, but unsuccessful, attempt to stand. This horse was nine months pregnant and was euthanised 3 h after discontinuation of inhalation anaesthesia. The third non-surviving horse (C19) was euthanised 14 h after standing due to progressive endotoxemia and bloody diarrhoea. Of the surviving colic horses four showed mild to moderate gait disturbances from the hind limbs during the study period. Clinical signs of myopathy (swollen, sore muscles) were not detected.

The healthy horses stood after one to four attempts (median 1.5). One healthy horse (H2) made several violent attempts to stand but without injuring itself. Two other horses were distressed during their attempts to stand and both of these showed symptoms of post-anaesthetic myopathy post anaesthesia; one had a slightly painful gracilis muscle (H10) and another developed a progressively worse triceps myopathy (H14). They were treated with flunixin after recovery. All healthy horses completed the study.

### Dialysate sampling

**Figure 2:**
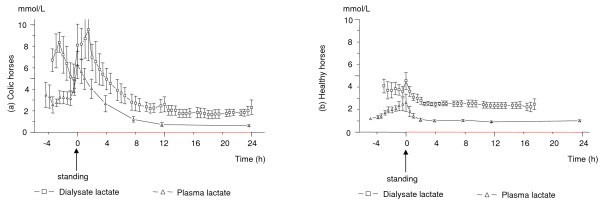
**Lactate concentrations in dialysate and plasma in colic and healthy horses**. The mean (± SEM) lactate concentrations in gluteal muscle dialysate and plasma in 8 colic horses (a) and in 10 healthy horses (b) during anaesthesia, in response to regaining the standing position (time 0) and up to 24 h after standing. Due to loss of the microdialysis catheter the number of dialysate samples decreases with time. At 10 h after standing there are results from 8 colic and from 5 healthy horses, and at 20 h after standing there are results from five colic horses but from no healthy horse.

**Figure 3:**
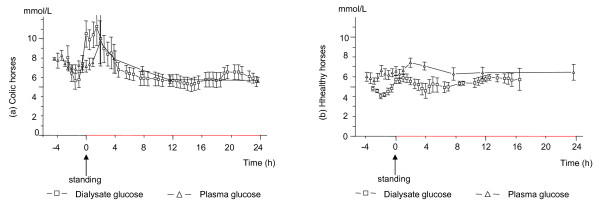
**Glucose concentrations in dialysate and plasma in colic and healthy horses**. The mean (± SEM) glucose concentrations in gluteal muscle dialysate and plasma in 8 colic (a) and 10 healthy horses (b) during anaesthesia, in response to regaining the standing position (time 0) and up to 24 h after standing. Due to loss of the microdialysis catheter the number of dialysate samples decreases with time. At 10 h after standing there are results from 8 colic and from 5 healthy horses, and at 20 h after standing there are results from five colic horses but from no healthy horse.

**Figure 4:**
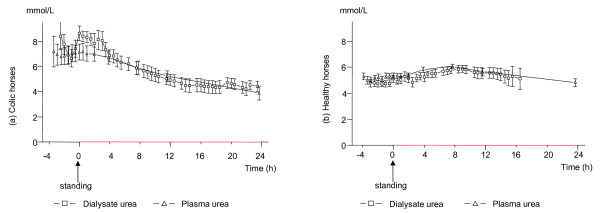
**Urea concentrations in dialysate and plasma in colic and healthy horses**. The mean (± SEM) urea concentrations in gluteal muscle dialysate and plasma in 8 colic horses (a), gluteal muscle dialysate urea concentrations in 10 healthy horses and plasma urea concentrations in 5 healthy horses (b), during anaesthesia, in response to regaining the standing position (time 0) and up to 24 h after standing. Due to loss of the microdialysis catheter the number of dialysate samples decreases with time. At 10 h after standing there are results from 8 colic and from 5 healthy horses, and at 20 h after standing there are results from five colic horses but from no healthy horse.

**Figure 5:**
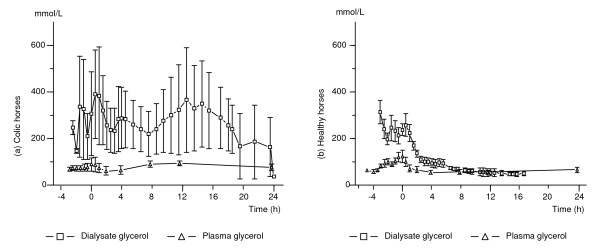
**Glycerol concentrations in dialysate and plasma in colic and healthy horses**. The mean (± SEM) glycerol concentration in plasma in 8 colic horses and in gluteal muscle dialysate in 4 colic horses (a), mean (± SEM) plasma and gluteal muscle dialysate glycerol concentrations in 10 healthy horses (b). The graphs show the changes during anaesthesia, in response to regaining the standing position (time 0) and up to 24 h after standing. Due to loss of the microdialysis catheter the number of dialysate samples decreases with time. At 10 h after standing there are results from 5 healthy horses.

Dialysate was successfully collected for a mean of 10 h 59 min and 20 h 43 min after recovery to standing in the healthy and the colic horses respectively. With time the membrane of the microdialysis catheters broke or the catheters were pulled out and at 20 h after standing there are results from five colic horses but from no healthy horse. Therefore, the mean levels at the end of the graphs in Figures 2, 3, 4, 5 were calculated from only a few samples.

### Lactate

The concentration of lactate was always higher in dialysate than in plasma in both groups (Figure 2a and 2b), but the concentration difference between dialysate and plasma varied greatly between groups, individuals and over time. In the colic horses the maximum dialysate-to-plasma difference occurred at time 0 (4.2 ± 1.3 mmol/L) while it occurred at 30 min after standing in the healthy horses (2.1 ± 0.3 mmol/L).

Dialysate lactate concentrations increased in all but one colic horse in response to the work of regaining the standing position and was significantly higher at 1 h (p = 0.02) and 2 h (p = 0.04) after standing compared to the end of anaesthesia. In the group of healthy horses there was no significant increase in dialysate lactate after regaining the standing position. The concentration of lactate in dialysate was significantly higher in the colic horses compared to the healthy horses at 1 h (C: 8.7 ± 1.8 and H: 3.1 ± 0.3 mmol/L, p = 0.02) and 2 h (C: 7.0 ± 1.2 and H: 2.8 ± 0.3 mmol/L, p = 0.04) after standing.

The general trends for the plasma lactate concentration changes were similar in colic and healthy horses but larger fluctuations were seen in the colic horses and the concentrations were higher in this group until 2 hours after standing. Plasma lactate increased from before anaesthesia to after one hour of anaesthesia in both colic horses (C: 2.2 ± 0.8 mmol/L to 3.4 ± 0.6 mmol/L, p < 0.001) and in the healthy horses (H: 0.5 ± 0.1 to 1.5 ± 0.1 mmol/L, p < 0.001). In the colic horses, the lactate concentration in plasma was significantly increased (p = 0.003) at 15 minutes after standing (6.2 ± 1.3 mmol/L), compared to the end of anaesthesia (3.1 ± 0.6 mmol/L) but decreased thereafter. In the healthy horses plasma lactate was significantly lower (p = 0.001) at two hours after standing (1.1 ± 0.1 mmol/L) compared to the end of anaesthesia (2.0 ± 0.2 mmol/L).

In the two most severely affected colic horses whose results are not included in the mean values (C8 and C14), lactate in both dialysate and plasma were above 15 mmol/L at all times and in C14 lactate in dialysate reached a maximum concentration of 42 mmol/L. In these horses, plasma lactate concentrations were 20.7 mmol/L and 15.4 mmol/L before anaesthesia and reached concentrations of 28.5 and 17.8 mmol/L at the end of anaesthesia. In horse C19, dialysate lactate increased post operatively, from 2.7 to 6.6 mmol/L when its condition deteriorated during the last hours before euthanasia. The healthy horse (H14) that developed a triceps myopathy had the highest concentrations of both dialysate and plasma lactate during anaesthesia (6 mmol/L and 4 mmol/L in dialysate and plasma respectively) and immediately after standing (8.1 mmol/L and 7.2 mmol/L in dialysate and plasma respectively) of all healthy horses. The concentrations decreased quickly thereafter.

### Pyruvate

Pyruvate in the dialysate was analysed in five colic horses, hence no statistical comparisons were performed on these data. The temporal changes in pyruvate basically followed the changes in lactate with an increase after standing, the maximum levels (0.3–0.5 mmol/L) being reached within 2–4 h after regaining the standing position and then a gradual decrease towards stable levels around 0.1 mmol/L.

#### The dialysate lactate-to-pyruvate ratio

The lactate-to-pyruvate ratio (La/Py ratio) reached its highest level at the beginning of sampling during anaesthesia with ratios varying from 38 to 75 and decreased thereafter. A short-lasting small increase was seen in association with the work of standing up. By 20 h after standing, in the three horses where samples still were obtained the ratio varied from 17 to 25. In the horse that was euthanised due to aggravating endotoxemia and diarrhoea 14 h after standing (C19), the La/Py ratio increased by more than 100% (from 15 to 43) during the last 2 h before euthanasia.

### Glucose

In the healthy horses the concentration of glucose was always lower in dialysate compared to that in plasma whereas in the colic horses the opposite situation was sometimes present, especially during anaesthesia and early after standing (Figure 3). In some colic horses the glucose levels in the dialysate exceeded that in plasma by 5–8 mmol/L.

In the colic horses dialysate glucose was increased during the first hours after standing compared to during anaesthesia (p < 0.01), whereas in the healthy horses there was no change over time. The concentration of dialysate glucose was higher in the colic horses than in the healthy horses, the difference being significant at time 0 (C: 10.5 ± 1.3 mmol/L and H: 5.7 ± 0.4 mmol/L, p = 0.01) and 1 h after standing (C: 10.4 ± 1.3 mmol/L and H: 5.9 ± 0.4 mmol/L, p = 0.001) and a near significant difference at 2 h after standing (C: 10.0 ± 2.8 mmol/L and H: 5.6 ± 0.4 mmol/L, p = 0.06).

The plasma glucose concentration was significantly higher in the colic than in the healthy horses during anaesthesia (p = 0.002) but not after standing. Plasma glucose did not change significantly after standing in either group, but tended to decrease over the following 12 h in the colic horses.

### Urea

The concentration of dialysate urea was significantly higher in the colic than in the healthy horses until at least 2 h after standing (p = 0.02) (Figure 4). In the colic horses dialysate urea increased significantly after standing (p = 0.003) at time 0 compared to the last sample during anaesthesia) and decreased slowly thereafter. The plasma urea level did not change significantly but the trend over time was similar to that of dialysate urea. The relationship between the dialysate and plasma concentrations varied over time and between individuals in the group of colic horses. Higher concentrations in the dialysate than in plasma were sometimes present during anaesthesia and in the early recovery-to-standing period whereas in the later samples, similar levels in the dialysate and plasma were seen. In the healthy horses urea concentrations remained stable showing no dialysate-to-plasma differences.

### Glycerol

In all healthy horses, the glycerol concentrations were always higher in dialysate than in plasma until immediately after or within a few hours after regaining the standing position, individual concentration differences being 2 to 10-fold. Thereafter, in those horses where dialysis continued to function, glycerol in dialysate was slightly lower or of similar concentration as in plasma (Figure 5b).

The plasma sample obtained in the healthy horses at 15 min after standing was significantly increased compared to all other sampling times (p = 0.04).

In the five colic horses in which dialysate glycerol was analysed, concentrations varied largely between individuals and over time (Figure 5a) and hence no statistical analysis was performed. The colic horse that died from pulmonary oedema and cardiovascular collapse during recovery (C8) had extremely high values (above 2200 mmol/L) during anaesthesia and early in recovery, but a decrease was seen in the last sample before the horse died. In this horse, the concentration of glycerol in plasma was approximately 50% of that in the dialysate.

## Discussion

The results show that with the microdialysis technique it was possible to study temporal changes in muscle lactate, glucose, glycerol, pyruvate and urea during anaesthesia and recovery in healthy and colic horses. Marked differences in the concentration levels between healthy and colic horses, as well as time-related changes were detected. The results from the healthy group of horses were more homogenous than those from the colic horses where large inter-individual differences were present reflecting different circulatory and metabolic status of the horses.

### The microdialysis technique

Microdialysis enabled nearly continuous monitoring of muscle interstitial concentrations of lactate, glucose, urea, glycerol and pyruvate in the horses studied. This technique offers unique opportunities to increase the knowledge about metabolism in the horse during various situations. It may not only be used in muscle but also in other tissues or body cavities where a dialysis catheter can be introduced [9,10,23]. Bed-side analysis may be performed using a commercial analyser (CMA 600, CMA/Microdialysis AB, Solna, Sweden) from the manufacturer of the microdialysis catheters.

Some difficulties were encountered in the present study using microdialysis in the freely moving horse; e.g. some catheters were accidentally pulled out or damaged when the horse moved or rubbed against the walls. A possible reason why the healthy horses lost their catheters at an earlier stage than the colic horses may be because they were moving around more in their stall. In the research setting, the risk of catheter loss would be reduced by inserting two or more catheters. In anaesthesia research, assisted recoveries and keeping the horses tied up when awake would probably also decrease this risk, but pose other problems instead, such as an increased risk of injury for the personnel.

An almost complete equilibrium with the true interstitial concentration is valuable since otherwise, different calibration methods have to be used to calculate this. With the long dialysis membrane and the low flow-rate used, the lactate, glycerol and urea concentrations in muscle dialysate were probably close to that in the interstitial space whereas glucose was slightly underestimated [3,8]. Further studies are necessary to find the exact perfusion rate where a 100% relative recovery of different metabolites is obtained in horses.

Some of the concentration differences that were found between dialysate and plasma may refer to the different methods for analysis and possibly to the effect of storage. However these factors should have affected the sample concentrations rather constantly over time and between groups why these factors are likely to have only minor influence on the results. A recently published study showed no statistical difference in metabolites when stored in microvials in -20C for 60 days [24].

### Metabolism

#### Lactate

The two horses with the highest concentrations of lactate in both dialysate and plasma did not survive. This finding agrees with earlier studies that found that the concentration of plasma lactate is a good prognostic indicator for survival in colic horses [17,19,25]. That lactate in dialysate is a useful parameter to follow in the postoperative period was also shown by the sudden concentration increase in dialysate in the colic horse that was euthanised 14 h after standing due to a deteriorating condition.

Traditionally, increased lactate production has been considered mainly as a marker for tissue ischaemia and anaerobic glycolysis but in the last decades, the role of lactate in different metabolic processes has been re-evaluated [26]. An increased rate of glycolysis due to sympathetic stimulation also results in increased lactate generation despite the presence of oxygen [11,27-29]. The high concentrations of lactate in plasma and dialysate seen in the colic horses probably resulted from a combination of accelerated glycolysis and anaerobic metabolism [30-32]. That anaerobic metabolism was contributing to energy production before and during anaesthesia in the colic horses was shown in a previous study by our group where the content of ATP in muscle was low and lactate high in several colic horses [20]. In the more severely ill colic horses, circulation is often compromised due to for example dehydration, electrolyte disturbances and endotoxemia, leading to poor peripheral perfusion. At the same time, many colic horses have an active colic behaviour where they walk and roll which increases their energy demands. To provide the muscle cells with energy, anaerobic metabolism must ensue. The relative contribution of the different causes for increased lactate production in the colic horses probably varied from case to case depending on the degree of stress and circulatory compromise.

Although lactate concentration changes in plasma mostly followed the changes in dialysate in both groups, the relationship between changes in dialysate and in plasma was not constant. In addition, with few exceptions, the plasma sample result underestimated the level in dialysate. These results confirm those from an earlier study [13]. This implies that by obtaining only plasma samples, certain events occurring in muscle will pass undiscovered [33].

An interesting pattern was seen in dialysate lactate during anaesthesia in several colic horses where an increase was followed by a decrease. This decrease could either reflect lactate being used as a substrate by the muscle cells [34] or by a slower rate of anaerobic glycolysis.

The greater increases seen in plasma and dialysate lactate in the colic horses compared to the healthy horses in response to regaining the standing position, and despite a visually good recovery, indicate that this period imposes more stress for the colic than the healthy horses. In most horses, a recovery requiring greater effort to stand was associated with greater increases in dialysate lactate, but not necessarily plasma lactate, compared to that in horses with a perfect and easy recovery.

#### Lactate-to-pyruvate ratio

Pyruvate, the precursor of lactate, and the La/Py ratio have gained increasing interest during the last decades as a means to distinguish between an increased rate of aerobic glycolysis, due for example to stress, and anaerobic production as the cause of the increased production of lactate [6,27,28,30,35,36]. If the lactate concentration increases but the ratio between lactate and pyruvate remains constant, there is no "excess" anaerobic production of lactate. In this situation the increased generation of lactate may not solely be the result of anaerobic metabolism but also a rapidly increased aerobic formation of pyruvate that can not enter the Krebs cycle [28,30]. Results from the five colic horses in the present study in which dialysate pyruvate was measured indicate that increased glycolysis also contributed to lactate production. This occurred especially in the period immediately after recovery to standing and is shown by increases in lactate in all horses while the La/Py ratio decreases in three out of the four horses that regained the standing position. The one of the three surviving horses (C13) that shows a remaining high La/Py ratio after standing experienced a very violent recovery (see below) while the other horses had acceptable to good recoveries with presumably less relative demands on anaerobic metabolism for the supply of energy.

#### Glucose

The finding that the plasma glucose concentrations in the healthy horses were slightly higher or similar to the concentrations in dialysate agrees with previous results in anaesthetised horses [13] and in human microdialysis studies [8,37]. Puzzling is that in several colic horses the glucose concentration was actually higher in dialysate than in plasma (Figure 3).

Blood flow may influence the concentration of glucose in dialysate [38-40] but does not explain the large discrepancy between plasma and dialysate concentrations (5–8 mmol/L). Since no healthy horse showed similarly higher glucose concentrations in dialysate compared to plasma, this phenomenon must relate to some factor unique for the systemically ill horses. One possibility for the increased concentrations of free glucose in the interstitial fluid may be related to a breakdown of muscle glycogen because this might result in some free glucose [41,42]. Muscle glycogen is used as a substrate during strenuous work, especially during short intensive bouts of exercise [43]. When the horses regain the standing position they perform similar type of work. Some of the increase observed in dialysate lactate after recovery to standing may partly have been due to an increased availability of glucose [41,44,45]. The increased concentrations in dialysate glucose during and after regaining the standing position in the colic horses may also depend on an increased sympathetic outflow and the anti-insulin effect of catecholamines and cortisol prohibiting transport of glucose from the interstitium into the cell and delaying the rate of utilisation of glucose [46].

#### Urea

The initially higher concentrations of dialysate and plasma urea in the colic horses compared to the healthy horses probably reflects decreased renal perfusion and excretion of urea depending on cardiovascular depression in the colic horses [18,47,48]. The subsequent decreasing concentration of urea over time in the colic horses accordingly is probably a result of improved circulation following correction of their primary condition.

**Figure 6:**
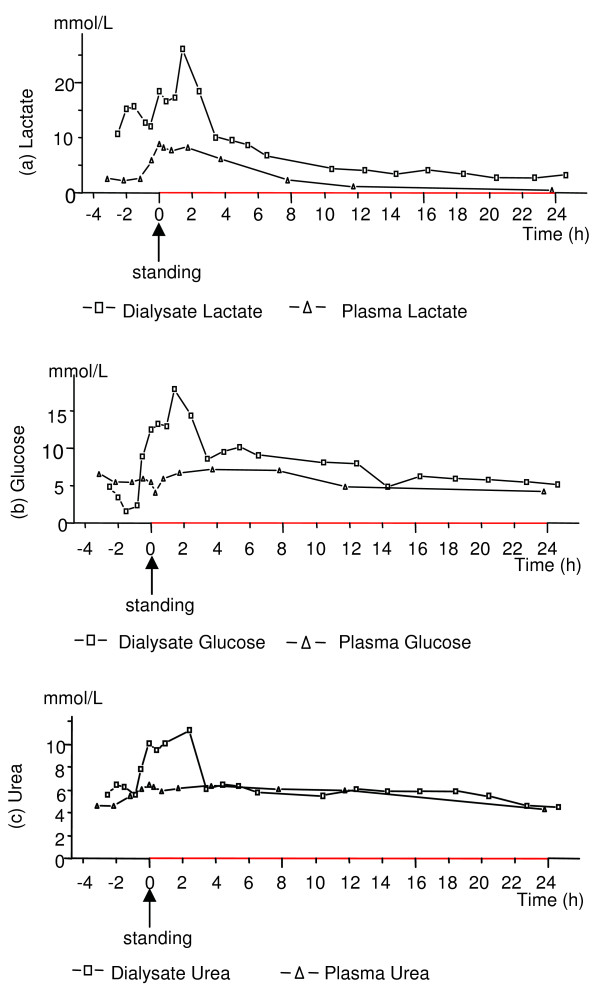
**Example of lactate, glucose, and urea changes in dialysate and plasma in a colic horse**. Concentrations of lactate (a), glucose (b), and urea (c) in plasma and gluteal muscle dialysate in one colic horse (C13) during anaesthesia, in response to standing (0) and until 24 h after regaining the standing position. In this horse, as in several other colic horses, higher concentrations of both urea and glucose in dialysate compared to plasma were observed during anaesthesia and in the early recovery period.

The transient increase in the dialysate urea level seen in the colic horses in response to regaining the standing position is difficult to explain. An increased recovery of urea has been referred to indicate an increased tissue blood flow [49] but since dialysis was performed at a very low flow rate that was identical in both healthy and colic horses, this metabolite would at least not be expected to be markedly higher in dialysate than in plasma as was the case in several colic horses (Figure 4, Figure 6c) but in no healthy horse. Changes in the plasma water content could possibly explain some of the increases in both glucose and urea in dialysate compared to plasma. However, as shown in the previous study by Edner et al. [20] the plasma protein concentration did not change over time during this period.

#### Glycerol

High initial concentrations of glycerol in dialysate after insertion of the catheter are usually considered to indicate cellular damage after introduction of the catheters [8,37,50]. A similar equilibration period as in the present study has been used by others and found to suffice [8,37,51], however dialysate glycerol had not stabilized in all horses by that time. Increased dialysate glycerol concentrations have also been found in response to increased intramuscular pressure in a porcine compartment syndrome model [35] and also during ischemia in humans [6,52]. Both of these processes may be present during anaesthesia in the horse [13,53-56]. Lipolysis of intramuscular stores of triglycerides occurs in humans in response to β-adrenergic stimulation [51] and this may be true also in the horse. The initially higher concentrations of glycerol in the dialysate compared to plasma in the healthy horses of the present study may therefore be an effect of increased intramuscular lipolysis. Results from a previous study suggest increased sympathetic stimulation during anaesthesia in healthy horses [13] since the concentration of plasma glycerol, free fatty acids and cortisol increased after induction of anaesthesia. Marked intramuscular lipolysis was probably the cause of the several-fold higher concentrations in dialysate compared to plasma during and after anaesthesia in the colic horses.

#### Case discussion

It is interesting to note that the colic horse (C13; Figure 6) that had the most violent recovery not only had very high concentrations of lactate in both dialysate (26 mmol/L) and plasma (8.9 mmol/L) after recovery to standing, but that this horse also had a very high concentration of lactate during anaesthesia in dialysate (15 mmol/L), however, not in plasma (2.5 mmol/L) (Figure 6a). The high La/Py ratio in this horse during anaesthesia and the first hours after standing indicates a significant anaerobic component during these periods. The results from a previous study [20] showed that during anaesthesia, this horse also had the lowest concentrations of serum potassium (2.5 mmol/L), high concentrations of plasma free fatty acids (above 600 mmol/L), and a muscle content of creatine phosphate that decreased markedly from the start to the end of anaesthesia (from 51 to 38 mmol/kg dry weight). These results together indicate that during anaesthesia this horse suffered from muscle hypoxia with consumption of energy sources. It is likely that those derangements in the muscle affected this horse's capacity to stand up smoothly.

Interestingly, similarly high interstitial concentrations of lactate during anaesthesia were seen in the healthy horse (H14) that also had a rough recovery and later developed a triceps myopathy. Anaesthesia was unremarkable with a mean blood pressure above 70 mmHg and an oxygen saturation > 99%. Since this horse also showed the highest glycerol concentrations in dialysate and plasma of the healthy horses during anaesthesia and no intramuscular changes in adenine nucleotides or creatine phosphate [20], this indicates that lipid metabolism was activated and the increase in lactate probably had other causes than increased anaerobic metabolism.

#### General discussion

The healthy horses consisted of a rather homogenous group of horses that were treated similarly and where the anaesthetic procedure alone affected muscle metabolism. In contrast, the group of colic horses was heterogeneous and metabolism was, in addition to anaesthesia, affected by different degrees of debilitation and medical treatment, and they also underwent surgery. It is therefore not always clear what was caused by anaesthesia and what was caused by the disease or surgery. However, the study aim was to investigate metabolic changes in colic horses undergoing anaesthesia compared with that in healthy horses undergoing anaesthesia.

Since the colic group was heterogeneous and small it is difficult to make statistical correlations between metabolite levels and different anaesthesia protocols, treatments, complications, speed of recovery etc. The metabolic state of the horse when entering anaesthesia largely influenced its metabolite levels both during anaesthesia and also early during recovery. A larger, more homogenous study group would have been preferable but was not possible at the time of the study. The metabolic parameters presented in the present study do not differ from the results of the study by Edner et al [20].

By studying metabolism in colic horses the metabolic processes will be further understood and will aid in improving the treatment and care of these horses.

## Conclusion

Microdialysis proved to be a valuable technique for the study of muscle metabolic events in the horse, since repeated sampling at a peripheral site and with minor intervention with the horse was possible. The results show that muscle production of lactate may be substantial especially in the colic horse and that the extent not always will be reflected by correspondingly high concentrations in plasma. The results also indicate that not only anaerobic lactate production but also other mechanisms such as an enhanced rate of aerobic glycolysis may contribute to the alterations seen in lactate concentrations during and after recovery from anaesthesia in colic horses. The metabolic response to regaining the standing position after anaesthesia was in general more severe in the colic horses than in the healthy horses. Further metabolic studies using microdialysis in the horse are encouraged.

## Competing interests

The authors declare that they have no competing interests.

## Authors' contributions

AE planned and carried out the study, performed most of the statistics and prepared the manuscript. BEG and GN participated in the design and carrying out of the study, interpretation of the results and helped to draft the manuscript. All authors read and approved the final manuscript.

## References

[B1] Ungerstedt U, Hallström A (1987). In vivo microdialysis- a new approach to the analysis of neurotransmitters in the brain. Life Sciences.

[B2] Ingvast-Larsson C, Appelgren LE, Nyman G (1992). Distribution studies of theophylline: microdialysis of theophylline in rat and horse. J Vet Pharmacol Ther.

[B3] Hagström-Toft E, Enoksson S, Moberg E, Bolinder J, Arner P (1997). Absolute concentrations of glycerol and lactate in human skeletal muscle, adipose tissue, and blood. Am J Physiol.

[B4] Groth L, Serup J (1998). Cutaneous microdialysis in man: effects of needle insertion trauma and anaesthesia on skin perfusion, erythema and skin thickness. Acta Dermato-Venereologica.

[B5] Ettinger SN, Poellmann CC, Wisniewski NA, Gaskin AA, Shoemaker JS, Poulson JM, Dewhirst MW, Klitzman B (2001). Urea as a recovery marker for quantitative assesment of tumor interstitial solutes with microdialysis. Cancer Res.

[B6] Ungerstedt J, Nowak G, Ericzon BG, Ungerstedt U (2003). Intraperitoneal microdialysis (IPM): a new technique for monitoring intestinal ischemia studied in a porcine model. Shock.

[B7] Rooyackers O, Thorell A, Nygren J, Ljungqvist O (2004). Microdialysis methods for measuring human metabolism. Curr Opin Clin Nutr Metab Care.

[B8] Rosdahl H, Hamrin K, Ungerstedt U, Henriksson J (1998). Metabolite levels in human skeletal muscle and adipose tissue studied with microdialysis at low perfusion flow. Am J Physiol.

[B9] Ungerstedt U (1991). Microdialysis- principles and applications for studies in animals and man. J Intern Med.

[B10] Jansson K, Ungerstedt J, Jonsson T, Redler B, Andersson M, Ungerstedt U, Norgren L (2003). Human intraperitoneal microdialysis: increased lactate/pyruvate ratio suggests early visceral ischaemia. A pilot study. Scand J Gastroenterol.

[B11] Luchette FA, Jenkins WA, Friend LA, Su C, Fischer JE, James JH (2002). Hypoxia is not the sole cause of lactate production during shock. J Trauma.

[B12] Edner A, Nyman G, Essén-Gustavsson B (2002). The relationship of muscle perfusion and metabolism with cardiovascular variables before and after detomidine injection during propofol-ketamine anaesthesia in horses. Vet Anaesth Analg.

[B13] Edner A, Essén-Gustavsson B, Nyman G (2005). Muscle metabolic changes associated with long-term inhalation anaesthesia in the horse analysed by muscle biopsy and microdialysis techniques. J Vet Med A Physiol Pathol Clin Med.

[B14] Manohar M, Gustafson R, Goetz TE, Nganwa D (1987). Systemic distribution of blood flow in ponies during 1.45%, 1.96%, and 2.39% end-tidal isoflurane-O2 anesthesia. Am J Vet Res.

[B15] Goetz TE, Manohar M, Nganva D, Gustafson R (1989). A study of the effect of isoflurane anaesthesia on equine skeletal muscle perfusion. Equine Vet J Suppl.

[B16] Serteyn D, Mottart E, Michaux C, Micheels J, Philippart C, Lavergne L, Guillon C, Lamy M (1986). Laser Doppler flowmetry: Muscular microcirculation in anaesthetized horses. Equine Vet J.

[B17] Moore JN, Owen RR, Lumsden JH (1976). Clinical evaluation of blood lactate levels in equine colic. Equine Vet J.

[B18] Parry BW, Anderson GA, Gay CC (1983). Prognosis in equine colic: a study of individual variables used in case assessment. Equine Vet J.

[B19] Ihler CF, Larsen JV, Skjerve E (2004). Evaluation of clinical and laboratory variables as prognostic indicators in hospitalised gastrointestinal colic horses. Acta Vet Scand.

[B20] Edner A, Nyman G, Essén-Gustavsson B (2007). Metabolism before, during and after anaesthesia in colic and healthy horses. Acta Vet Scand.

[B21] Rosdahl H, Ungerstedt U, Henriksson J (1997). Microdialysis in human skeletal muscle and adipose tissue at low flow rates is possible if dextran-70 is added to prevent loss of perfusion fluid. Acta Physiol Scand.

[B22] Lowry OH, Passonneau JV (1973). A flexible system of enzymatic analysis.

[B23] Delgado JM, DeFeudis FV, Roth RH, Ryugo DG, Mitruka BM (1972). Dialytrode for long term intracerebral perfusion in awake monkeys. Arch Int Pharmacodyn.

[B24] Abrahamsson P, Johansson G, Åberg A-M, Haney M, Winsö O (2008). Optimised sample handling in association with use of the CMA 600 analyser. J Pharm Biomed Anal.

[B25] Parry BW, Anderson GA, Gay CC (1983). Prognosis in equine colic: a comparative study of variables used to assess individual cases. Equine Vet J.

[B26] Gladden LB (2004). Lactate metabolism: a new paradigm for the third millenium. J Physiol.

[B27] James JH, Luchette FA, McCarter FD, Fischer JE (1999). Lactate is an unreliable indicator of tissue hypoxia in injury or sepsis. Lancet.

[B28] Gore DC, Jahoor F, Hibbert JM, DeMaria EJ (1996). Lactic acidosis during sepsis is related to increased pyruvate production, not deficits in tissue oxygen availability. Ann Surg.

[B29] Wolfe RR, Martini WZ (2000). Changes in intermediary metabolism in severe surgical illness. World J Surg.

[B30] Huckabee WE (1958). Relationships of pyruvate and lactate during anaerobic metabolism. III. Effect of breathing low-oxygen gases. J Clin Invest.

[B31] Larsson J, Hultman E (1979). The effect of long-term, arterial occlusion on energy metabolism of the human quadriceps muscle. Scand J Clin Lab Invest.

[B32] Harris K, Walker PM, Mickle DA, Harding R, Gatley R, Wilson GJ, Kuzon B, McKnee N, Romaschin AD (1986). Metabolic response of skeletal muscle to ischaemia. Am J Physiol.

[B33] Müller M, Schmid R, Nieszpaur-Los M, Fassolt A, Lönnroth P, Fasching P, Eichler HG (1995). Key metabolite kinetics in human skeletal muscle during ischaemia and reperfusion: measurement by microdialysis. Eur J Clin Invest.

[B34] Gladden LB (2000). Muscle as a consumer of lactate. Med Sci Sports Exrec.

[B35] Ungerstedt J, Goiny M, Ungerstedt U (2002). Acute compartment syndrome in a pig model monitored with microdialysis. 3rd Scandinavian Microdialysis User Symposium; Bålsta, Sweden.

[B36] Setälä LP, Korvenoja EM-L, Härmä MA, Alhava EM, Uusaro AV, Tenhunen JJ (2004). Glucose, lactate and pyruvate response in an experimental model of microvascular flap ischemia and reperfusion: a microdialysis study. Microsurgery.

[B37] Fuchi T, Rosdahl H, Hickner RC, Ungerstedt U, Henriksson J (1994). Microdialysis of rat skeletal muscle and adipose tissue: dynamics of the interstitial glucose pool. Acta Physiol Scand.

[B38] Rosdahl H, Ungerstedt U, Jorfeldt L, Henriksson J (1993). Interstitial glucose and lactate balance in human skeletal muscle and adipose tissue studied by microdialysis. J Physiol.

[B39] Rosdahl H (1998). Microdialysis sampling from skeletal muscle and adipose tissue with special reference to the effects of insulin on tissue blood flow and glucose metabolism. Doctoral thesis.

[B40] Hickner RC, Rosdahl H, Borg I, Ungerstedt U, Jorfeldt L (1992). The ethanol technique of monitoring local blood flow changes in rat skeletal muscle: implications for microdialysis. Acta Physiol Scand.

[B41] Newsholme EA, Leech AR, Eds (1983). Biochemistry for the medical sciences.

[B42] Essén B, Kaijser L (1978). Regulation of glycolysis in intermittent exercise in man. J Physiol.

[B43] Gottlieb M (1989). Muscle glycogen depletion patterns during draught work in Standardbred horses. Equine Vet J.

[B44] Kerckhoffs DAJM, Arner P, Bolinder J (1998). Lipolysis and lactate production in human skeletal muscle and adipose tissue following glucose ingestion. Clin Sci (Lond).

[B45] Del Canale S, Fiaccadori E, Ronda N, Söderlund K, Antonucci C, Guariglia A (1986). Muscle energy metabolism in uremia. Metabolism.

[B46] Lager I (1991). The insulin-antagonistic effect of the counterregulatory hormones. J Intern Med Suppl.

[B47] Parry BW (1987). Use of clinical pathology in evaluation of horses with colic. Vet Clin North Am Equine Pract.

[B48] Svendsen CK, Hjortkjaer RK, Hesselholt M (1979). Colic in the horse. A clinical and clinical chemical study of 42 cases. Nord Vet Med.

[B49] Lundberg G, Olofsson P, Ungerstedt U, Jansson E, Sundberg CJ (2002). Lactate concentrations in human skeletal muscle biopsy, microdialysate and venous blood during dynamic exercise and under blood flow restriction. Pflugers Arch.

[B50] Henriksson J (1999). Microdialysis of skeletal muscle at rest. Proc Nutr Soc.

[B51] Hagström-Toft E, Enoksson S, Moberg E, Bolinder J, Arner P (1998). beta-Adrenergic regulation of lipolysis and blood flow in human skeletal muscle in vivo. Am J Physiol.

[B52] Östman B, Michaelsson K, Rahme H, Hillered L (2004). Torniquet-induced ischemia and reperfusion in human skeletal muscle. Clin Orthop Relat Res.

[B53] Lindsay WA, McDonell WN, Bignell W (1980). Equine postanesthetic forelimb lameness: Intracompartmental muscle pressure changes and biochemical patterns. Am J Vet Res.

[B54] Steffey EP, Lindsay WA, Mc Donell WN, Bignell W (1980). Equine postanesthetic forelimb lameness: Intracompartmental muscle pressure changes and biochemical patterns. Am J Vet Res.

[B55] Serteyn D, Mottart E, Deby C, Deby-Dupont G, Pincemail G, Philippart C, Lamy M (1990). Equine postanaesthetic myositis: a possible role for free radical generation and membrane lipoperoxidation. Res Vet Sci.

[B56] Serteyn D, Pincemail G, Mottart E, Caudron I, Deby C, Deby-Dupont G, Philippart C, Lamy M (1994). Approche directe pour la mise en évidence des phénomènes radicalaires lors myopathie postanesthésique équine: étude peliminaire. Can J Vet Res.

